# Effect of Metformin on Viability, Morphology, and Ultrastructure of Mouse Bone Marrow-Derived Multipotent Mesenchymal Stromal Cells and Balb/3T3 Embryonic Fibroblast Cell Line

**DOI:** 10.1155/2015/769402

**Published:** 2015-04-30

**Authors:** Agnieszka Śmieszek, Aleksandra Czyrek, Katarzyna Basinska, Justyna Trynda, Aneta Skaradzińska, Anna Siudzińska, Monika Marędziak, Krzysztof Marycz

**Affiliations:** ^1^Electron Microscopy Laboratory, The Faculty of Biology and Animal Science, University of Environmental and Life Sciences, Kożuchowska 5b Street, 50-631 Wroclaw, Poland; ^2^Wrocławskie Centrum Badań EIT+, Stablowicka 147 Street, 54-066 Wroclaw, Poland; ^3^Department of Experimental Oncology, Ludwik Hirszfeld Institute of Immunology and Experimental Therapy, Polish Academy of Sciences, 53-114 Wroclaw, Poland; ^4^Department of Biotechnology and Food Microbiology, Faculty of Food Science University of Environmental and Life Sciences, Chelmonskiego 37/41, 51-630 Wroclaw, Poland

## Abstract

Metformin, a popular drug used to treat diabetes, has recently gained attention as a potentially useful therapeutic agent for treating cancer. In our research metformin was added to *in vitro* cultures of bone marrow-derived multipotent mesenchymal stromal cells (BMSCs) and Balb/3T3 fibroblast at concentration of 1 mM, 5 mM, and 10 mM. Obtained results indicated that metformin negatively affected proliferation activity of investigated cells. The drug triggered the formation of autophagosomes and apoptotic bodies in all tested cultures. Additionally, we focused on determination of expression of genes involved in insulin-like growth factor 2 (IGF2) signaling pathway. The most striking finding was that the mRNA level of IGF2 was constant in both BMSCs and Balb/3T3. Further, the analysis of IGF2 concentration in cell supernatants showed that it decreased in BMSC cultures after 5 and 10 mM metformin treatments. In case of Balb/3T3 the concentration of IGF2 in culture supernatants decreased after 1 and 5 mM and increased after 10 mM of metformin. Our results suggest that metformin influences the cytophysiology of somatic cells in a dose- and time-dependent manner causing inhibition of proliferation and abnormalities of their morphology and ultrastructure.

## 1. Introduction

Metformin is a common drug used worldwide in the treatment of diabetes mellitus. It belongs to the group of biguanidine drugs, among which it has the best safety profile [1]. The general systemic effect of metformin involves the reduction of glucose concentration and increased insulin sensitivity. However, mounting evidence indicates that the range of metformin action may be significantly wider, and thus the application of metformin may open new perspectives in the treatment of various medical conditions [2, 3]. In cell culture, metformin inhibits the proliferation of a range of cancer cells, including breast [4–6], oral cavity [7], pancreas [8], and ovarian cells [9]. Effectiveness of this agent as an anticancer drug is associated not only with its cytostatic properties but also with proapoptotic action in tumor cells [7, 10, 11].

Metformin is also assigned to the conceptual group of drugs, known as calorie restriction mimetics (CRM). It has been demonstrated that calorie restriction is a very effective way of increasing the lifespan by reducing morbidity and mortality in mice with tumors [12]. The key signaling pathways underlying the antiaging effects of metformin or other CRM drugs have not been fully explored. It seems that metformin affects endocrine regulatory systems and insulin-like growth factors [13]. Signaling pathway of insulin-like growth factors (IGF) regulates cell proliferation, differentiation, aging, and life span; thus its role is principal for the development of the organism and has remained unchanged during evolution [14]. IGF2, together with the H19 gene, form an imprinted tandem both in humans and in mice that plays an important role not only during embryonic development but also during the proliferation of stem cells residing in adult tissues [14, 15].

Bone marrow provides a niche for various populations of stem cells, the interplay of which is essential for body homeostasis. Biology of the bone marrow-derived multipotent mesenchymal stromal cells (BMSCs) is continuously being studied. Their potential for self-renewal as well as high phenotypic plasticity, manifested by the ability to differentiate into bone, cartilage, or adipose tissue, is extremely important in terms of regenerative medicine [16]. Mesenchymal stromal stem cells (MSCs), due to a high phenotypic and cellular plasticity, are a suitable model for* in vitro* assessment of various biological and chemical agents [17]. Additionally, evaluation of alterations in MSC morphology provides valuable information that reflects complex biological processes controlled by the interactions between the cytoskeleton and the extracellular environment [18].

The properties of self-renewal and differentiation of stem cells might be regulated by octamer-binding protein 4 (Oct-4), a transcription factor crucial for embryonic development [19]. The expression of Oct-4 was reported in bone marrow-derived stromal cells, which confirms high phenotypic plasticity of these cells [20]. Impairment of the proliferation potential of mesenchymal stem cells may account for regenerative potential deficiency of the organism. Mesenchymal stem cells seem to participate in the process of bioactive stroma formation [21] and affect the biological properties of surrounding tissues. Due to the fact that metformin increases glucose uptake in connective and embryonic tissues [22], their effect on proliferative activity of BMSCs and other cells of connective tissue, such as fibroblasts, should be considered. In the present work, we have evaluated the effect of metformin* in vitro* using murine primary cultures of bone marrow-derived multipotent mesenchymal stromal cells and Balb/3T3 fibroblast cell line.

We have investigated the effect of metformin in cell cultures at doses cytotoxic for cancer cells [4, 5, 7–9]. Our objective was to determine how different concentrations of metformin affect the physiology of stromal cells. The analysis included BMSC and Balb/3T3 proliferation activity assays and evaluation of the morphology and ultrastructure of cells investigated. We have also aimed to determine the expression of IGF signaling components (IGF2, IGF2R, and H19) as well as the expression of Oct-4.

## 2. Materials and Methods

All reagents used in this experiment were purchased from Sigma-Aldrich (Poland), unless indicated otherwise.

### 2.1. Ethical Approval

The study was conducted with the approval of the Bioethics Committee, as stated by the Second Local Bioethics Committee at the Department of Biology and Animal Breeding, Wroclaw University of Environmental and Life Sciences, Wroclaw, Chelmonskiego 38C, Poland (Dec. number 177/2010 of 11.15.2010).

### 2.2. Cell Population

Two types of mouse cells were used in the experiments: multipotent stromal cells (BMSCs) derived from bone marrow (primary cultures) and Balb/3T3 embryonic fibroblasts (cell line obtained from the Institute of Immunology and Experimental Therapy, Polish Academy of Sciences).

### 2.3. Isolation of BMSCs

Bone marrow-derived multipotent mesenchymal stromal cells (BMSCs) were isolated from twelve 4-week-old C57BL/6 mice (Animal Vivarium Wroclaw Medical School, Poland). Femurs were collected directly after euthanasia of the animal and placed in a sterile Hanks' balanced salt solution (HBSS). Cells were isolated from the bone marrow by flushing with an insulin syringe U-40 (29G X 1/2′′ needle) filled with HBSS. Cell suspension was transferred into falcon tube and centrifuged at 300 ×g for 4 minutes. For FACS analysis, bone marrow cells were lysed in BD lysing buffer (BD Biosciences, San Jose, CA, USA) for 15 min at room temperature and washed twice in phosphate-buffered saline (PBS). For cell culture, pellets were resuspended in Dulbecco's Modified Eagle's Medium (DMEM) containing Ham's F-12 nutrient mixture supplemented with 10% of foetal bovine serum (FBS) and transferred to culture flasks.

### 2.4. Characterization of BMSCs Phenotype: Flow Cytometry

Bone marrow cell suspensions, isolated by flushing femurs and tibia, were lysed in BD lysing buffer (BD Biosciences, San Jose, CA, USA) for 15 min at room temperature and washed twice in phosphate-buffered saline (PBS). The cells were subsequently stained for Sca-1 antigen and hematopoietic lineage markers (Lin) for 30 min in medium containing 2% foetal bovine serum. The following anti-mouse antibodies (BD Pharmingen) were used for staining: Sca-1 (FITC, clone D7), B220 (PE, clone RA3-6B2), T-cell receptor-*β* (PE, clone H57-597), T-cell receptor-*γδ* (PE, clone GL3), CD11b (PE, clone M1/70), Ter119 (PE, clone TER-119), and Gr-1 (PE, clone RB6-8 C5). Sca-1+/Lin− cells were isolated by a multiparameter, live-cell sorting (INFLUX, BD).

To phenotype BMSC cell surface antigens, Sca-1+/Lin− cells were stained using the following: CD31 (APC, clone 390), CD45 (APC-Cy7, clone 30-F11), CD51 (biotin, clone RMV-7 with streptavidin conjugated to PE-Cy5), CD73 (FITC, clone B5), CD90 (PB, clone 53-2.1), and CD105 (PE, MJ7/18). All monoclonal antibodies (mAbs) were added at saturating concentrations and the cells were incubated for 30 minutes on ice, washed twice, resuspended in staining buffer at a concentration of 5 × 10^6^ cells per millilitre, and analyzed using an LSR II (BD Biosciences, Mountain View, CA, http://www.bdbiosciences.com/). Anti-mouse mAbs were purchased from BD Pharmingen (San Diego, CA, http://www.bdbiosciences.com/).

### 2.5. Determination of Multipotent Character of BMSCs

Adipogenic and osteogenic differentiation of BMSCs was induced using commercial kits (StemPro, Life Technologies). Stimulation toward adipocytes lasted 14 days, while osteogenesis was induced during a 21-day period. To evaluate adipogenic and osteogenic differentiation, two specific staining methods were used, that is, Oil-Red O for the detection of neutral lipid deposits and Alizarin red for calcium deposits. Preparations were analyzed using an Axio Observer A1 inverted microscope (Carl Zeiss, Jena, Germany). Documentation was made using Canon PowerShot camera.

### 2.6. Propagation of Cells

Cultures were maintained at 37°C in a humidified atmosphere of 5% CO_2_ and 95% air. Primary and subsequent cultures of BMSCs were propagated in Dulbecco's Modified Eagle's Medium (DMEM) with Ham's F-12 nutrient mixture, while Balb/3T3 were maintained in DMEM containing 4500 mg/L of glucose. All culture media were supplemented with 10% of FBS and 1% of antibiotics (penicillin and streptomycin). Medium was changed every two days. The passage of cells was performed at 80–90% confluence. Prior to the experiment, the cells were passaged three times using trypsin solution (TrypLE; Life Technologies) according to the manufacturers' instruction.

### 2.7. Cultures with Metformin

Metformin (Metformax 850; Teva Pharmaceuticals, Poland) was grinded with a mortar and dissolved in the culture medium at the following concentrations: 1 mM, 5 mM, and 10 mM. Nontreated cells served as a control for comparison with the test cultures. For the analysis of proliferation, morphology, ultrastructure, and gene expression both BMSCs and Balb/3T3 were inoculated into 24-well plates, while measurements of DNA synthesis were performed in cultures propagated in 96-well plates. Initial concentration of cells in 24-well dishes was 3 × 10^4^ per well. The cells were inoculated in a 0.5 mL volume of culture medium per well. Seeding density of cells in 96-well plates was 5 × 10^3^. Cells were inoculated in 0.1 mL of complete growth medium per well. The first dose of the drug tested was added to the medium after 24 hours, when adhesion and spreading of cells were observed on the plate surface. During cell propagation medium was changed every day. The experiment was performed in three independent replicates.

### 2.8. Proliferation of Cells

#### 2.8.1. Analysis Using Resazurin Assay

Cell viability was evaluated after 24, 48, and 72 hours using resazurin-resorufin system. To perform the assay, medium was removed and replaced with a medium containing 10% of the dye. Cells were incubated in a CO_2_ incubator for 2 hours and then the supernatants were collected and transferred into the 96-well microplate reader (Spectrostar Nano, BMG Labtech). Supernatants after BMSCs and Balb/3T3 cultures were derived from three independent experiments. The absorbance of the supernatants was measured spectrophotometrically at a wavelength of 600 nm for resazurin and 690 nm as a reference wavelength. Each test included a blank containing complete medium without cells.

#### 2.8.2. Measurement of DNA Synthesis: BrdU Assay

DNA synthesis was assessed by measuring the incorporation of 5-bromo-2-deoxyuridine (BrdU) into cellular DNA. Proliferation of cells was analyzed three times independently after 24, 48, and 72 hours of the experiment. The assay was carried out using BrdU Cell Proliferation ELISA Kit, based on the protocol provided by the manufacturer (Abcam). Briefly, cultures were treated with BrdU and incubated overnight at 37°C in a humidified atmosphere. After incubation with BrdU, cells were fixed and DNA was denatured using a Fixing Solution provided by the manufacturer. BrdU incorporation was detected using anti-BrdU monoclonal antibody. Incubation of cells with specific antibody was performed at room temperature and lasted for 1 hour. Goat anti-mouse IgG conjugated with horseradish peroxidase (HRP) was used as secondary antibody. Incubation with secondary antibody was performed at room temperature for 30 minutes. Color reaction was developed using 3,3′,5,5′-tetramethylbenzidine (TMB) as substrate and stopped after 30 minutes. Incubation with substrate was performed at room temperature, avoiding exposition to excessive light. Signal intensity was measured with a spectrophotometer microplate reader (Spectrostar Nano, BMG Labtech) at a wavelength of 450/550 nm.

#### 2.8.3. Morphology of Cells

The morphology of the studied cells was evaluated with an epifluorescent microscope (Zeiss, Axio Observer A.1) and scanning electron microscope (SEM, Zeiss Evo LS 15). The analysis of morphology was performed after 48 h of the experimental culture in 24-well plates. Preparation of cells for fluorescence microscopy was as follows: cells were (i) washed three times using HBSS, 1 minute each wash; (ii) fixed in 4% ice cold paraformaldehyde, overnight at 4°C; (iii) washed (as described above); (iv) permeabilized for 15 minutes with 0.1% Triton X-100, at room temperature; (v) washed (as described above); (vi) stained with atto-488-labeled phalloidin (1 : 800) for 30 minutes in the dark at room temperature; and (vii) counterstained using diamidino-2-phenylindole (DAPI; 1 : 1000), for 5 minutes at room temperature as described previously [23]. In addition, propidium iodide staining was performed to detect dead cells in the population. Propidium iodide was diluted in PBS (1 : 1000). Cultures were incubated with a dye for 15 minutes at 37°C. Images of stained cultures were captured using a PowerShot Camera (Canon). SEM analysis was as follows: cell cultures were (i) fixed in 2.5% glutaraldehyde in DMEM, (ii) rinsed with HBSS, (iii) dehydrated in a graded ethanol series (from 50% to 100%, increasing 10% at each step), (iv) air-dried for 30 minutes at room temperature, and (v) coated with gold particles using 300-second program (Edwards, Scancoat six). Prepared samples were imaged using SE1 detector at 10 kV filament tension (SEM, Zeiss Evo LS 15) and 500x and 5000x magnification as described previously [24, 25].

#### 2.8.4. Ultrastructure of Cells

Ultrastructure analysis of cells was performed by using a scanning transmission electron microscope (TEM, Zeiss Evo LS 15) as described previously [26]. The cells were fixed overnight at 4°C in 2.5% glutaraldehyde in DMEM. After fixing, the cells were centrifuged at 2000 ×g for 10 minutes and rinsed with PBS (0.1 M, pH = 7.0) for 30 minutes at room temperature as described previously. Then the cells were centrifuged once again, using settings provided above. The resulting pellets were incubated with 1% osmium tetroxide in PBS for 2 hours. Next, cells were washed using 0.1 M PBS and centrifuged. After this procedure, cells were dehydrated in a graded acetone series (30–100%) and embedded in Agar Low Viscosity Resin Kit (Agar Scientific Ltd., Stansted, Essex, UK). Ultrathin sections (80 nm) of the specimens were collected on copper grids. Cells were contrasted with uranyl acetate (30 minutes incubation) and lead citrate (15 minutes incubation). The cells were observed with the TEM detector, at 10 kV filament tension.

#### 2.8.5. Analysis of Gene Expression: Real-Time Reverse-Transcription Polymerase Chain Reaction (qRT-PCR)

Cells were rinsed twice using HBSS after 48 h culture and then homogenized using 0.8 mL of TRI Reagent. Total RNA was isolated according to a single-step method described by Chomczynski and Sacchi [27]. The resulting samples were diluted in DEPC-treated water. Quantity and quality of total RNA was determined using nanospectrometer (WPA Biowave II). Traces of genomic DNA (gDNA) were digested with DNase I RNase-free kit (Thermo Scientific). Each reaction contained 200 ng of total RNA. Complementary DNA (cDNA) was obtained in the reaction with Moloney Murine Leukemia Virus Reverse Transcriptase (M-MLV RT) and oligo(dT)15 primers (Verte KIT oligo(dT)15, Novazym). Both RNA purification and cDNA synthesis were performed in accordance with the manufacturers' instructions using a T100 Thermo Cycler (Bio-Rad). Sequences of the primers used in the amplification are listed in Table 1. Quantitative RT-PCR was carried out in a total volume of 20 *μ*L using SensiFast SYBR & Fluorescein Kit (Bioline). PCR mixture contained 20% of cDNA. Concentration of primers in each reaction was 500 nM. The following cycling conditions were applied: 95°C for 2 minutes, followed by 45 cycles of 95°C for 5 s, annealing temperature gradient for 10 s, and 72°C for 5 s with a single fluorescence measurement. To determine the specificity of the PCR products, analysis of the dissociation curve of amplicons was performed. Melting curve was determined with a program ramped up from 65 to 95°C at a heating rate of 0.2°C/s and continuous measurement of the fluorescence. The value of the threshold cycle (Ct) was used to calculate the fold change in relation to the expression of housekeeping gene, beta-2 microglobulin (*β*2m). Real-time PCR was performed using CFX Connect Real-Time PCR Detection System (BioRad).

#### 2.8.6. Enzyme-Linked Immunosorbent Assay (ELISA)

The supernatants of the cultures were analyzed by ELISA to determine the concentration of secreted IGF2. Supernatants were collected after 48 hours of cell propagation from control and experimental cultures. Aliquots were kept at −20°C until ELISA analysis. Marker protein was assayed using specific mouse IGF-II ELISA kit (DuoSet ELISA Development kit; R&D Systems, Poland). Before each assay, all samples were briefly centrifuged and twofold diluted. The substrate for peroxidase used in ELISA was 3,3′,5,5′-tetramethylbenzidine (TMB), and the reaction was stopped with 2 N sulfuric acid (H_2_SO_4_). Readouts were conducted at a wavelength of 450 nm using a spectrophotometer (BMG Labtech).

#### 2.8.7. Statistical Analysis

Normality of the population data was determined using the Shapiro-Wilk test, while equality of variances was assessed by Levene's test. Differences between groups were determined using one- or two-way analysis of variance (ANOVA). Statistical analysis was performed with STATISTICA 10.0 software (StatSoft, Inc., Statistica for Windows, Tulsa, OK, USA). Differences with a probability of *p* < 0.05 were considered significant.

## 3. Results

### 3.1. Phenotypic Characterization of BMSCs and Their Multipotent Properties

**Figure 1:**
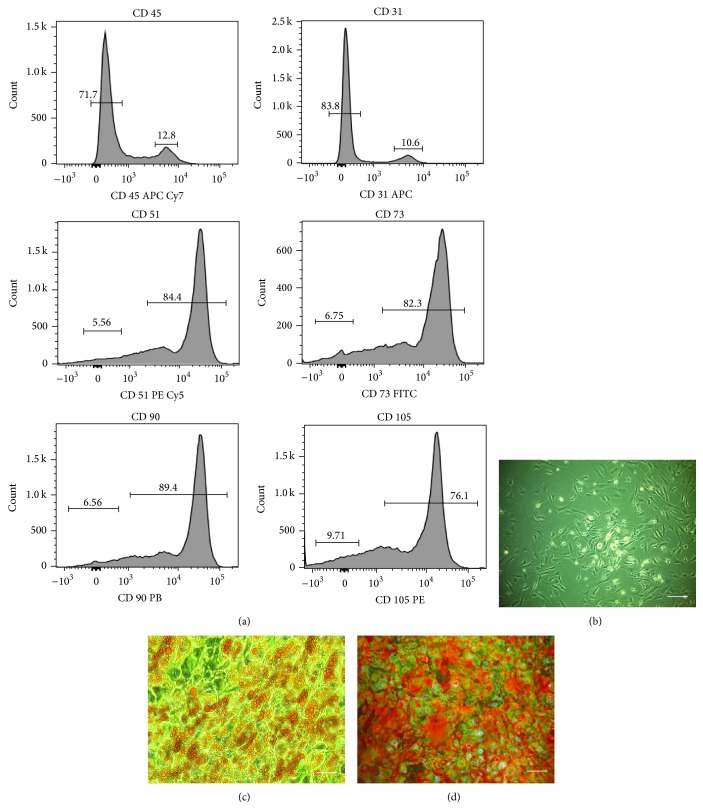
Characterization of BMSC phenotype and determination their multipotency. Phenotype of BMSCs was determined by flow cytometry. The analysis revealed that BMSCs isolated by the described method were negative for CD45 and CD31. In addition, the cells were strongly positive for CD51, CD73, CD90, and CD105 (panel (a)). Morphology of BMSC culture in standard (b), adipogenic (c), and osteogenic conditions (d). Specific stainings were carried out to determine the BMSC differentiation to adipocytes and osteocytes. Oil-Red O staining was used to detect lipid droplet formation during adipogenic differentiation (c). Alizarin red staining was used to detect calcium deposition during osteogenic differentiation (d). Images of differentiated cultures were captured at 100x magnification (scale bar = 200 *μ*m).

The analysis showed that BMSCs, isolated according to the method described, expressed markers specific for multipotent mesenchymal stromal cells, that is, CD51, CD73, CD90, and CD105. Cells were negative for hematopoietic marker, that is, CD45 and endothelial marker CD31. (Figure 1(a)). Additionally, multipotent nature of cells was confirmed by their ability to differentiate into bone and adipocyte precursors. In contrast to the cells cultured in standard conditions (Figure 1(b)), specific staining showed that stimulation of BMSCs with the adipogenic medium promotes the formation of lipid droplets, while induction of osteogenesis resulted in the formation of calcium-rich deposits (Figures 1(c) and 1(d)).

### 3.2. Proliferation of Cells

**Figure 2:**
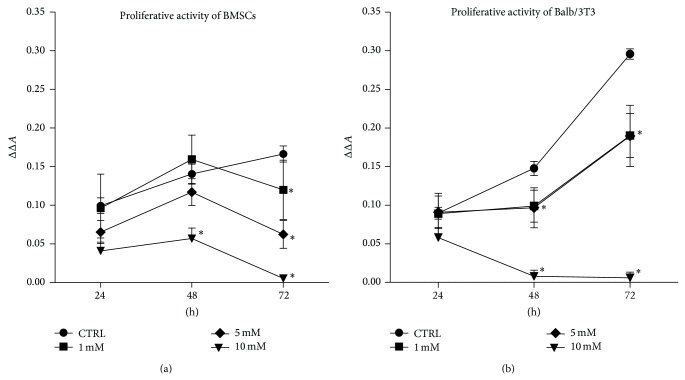
Influence of metformin on the proliferative activity of BMSCs (a) and mouse embryonic fibroblast cell line Balb/3T3 (b). Proliferation of control cultures was compared to the cultures propagated with metformin. (a) No difference in the proliferation rate of BMSCs was recorded after 24 and 48 hours (*p* > 0.05) when cells were cultured with the addition of 1 mM and 5 mM metformin. A significant decrease in the proliferative activity of BMSCs treated with 1 mM and 5 mM concentrations was noticed after 72 hours. The addition of 10 mM metformin to the BMSC culture significantly reduced the proliferation of BMSCs from the second day of culture. (b) Proliferative activity of Balb/3T3 in experimental cultures decreased significantly after 48 hours. Statistically significant differences in the experimental and control culture of Balb/3T3 were also observed after 72 hours of propagation. An asterisk (∗) indicates a statistically significant difference (*p* < 0.01). Significance was determined by two-way ANOVA test. Each test included a blank containing complete medium without cells. The *x*-axis refers to the time of cell propagation, while ΔΔ*A* mark on *y*-axis refers to the difference between absorbance read at 600 nm and 690 nm, including blank sample.

**Figure 3:**
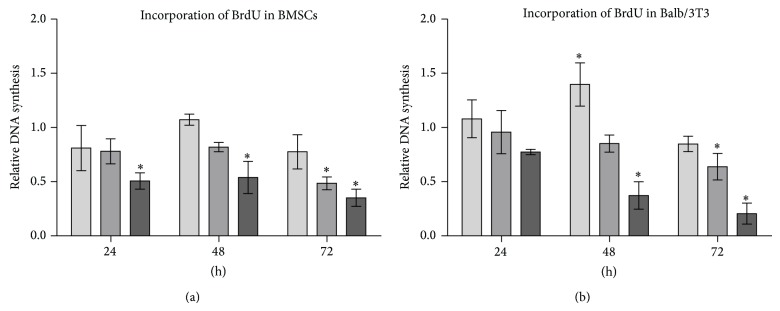
Results of BrdU incorporation assay. (a) Cytotoxic effect of 10 mM metformin was recorded starting from 24th hour of BMSC culture. Metformin at 5 mM concentration significantly reduced DNA synthesis in BMSC cultures after 72 hours of treatment. Metformin at the lowest investigated concentration did not affect DNA synthesis. (b) Increase of DNA synthesis was observed in Balb/3T3 after 48 hours of culture with 1 mM metformin. Cytotoxic effect of metformin was prominent after 72 hours of culture, especially when Balb/3T3 were treated with metformin at 5 and 10 mM doses. Fold change in DNA synthesis was calculated by comparing BrdU signals of metformin-treated cells to that of the control culture, to which a value of 1 was assigned. An asterisk (^∗^) indicates a statistically significant difference (*p* < 0.05). Significance was determined by two-way ANOVA test (*p* < 0.05).

Cellular proliferative activity of both of BMSCs as well as mouse embryonic fibroblast cell line Balb/3T3 was evaluated after 24, 48, and 72 hours of culture (Figures 2 and 3). The results of resazurin-based assay revealed that exponential pattern of cell growth was observed only in the control cultures of BMSCs and Balb/3T3. The proliferation of BMSCs treated with metformin at a concentration of 1 mM and 5 mM was comparable with the control culture until 48 hours of propagation. A slight, however, not statistically significant, increase in the rate of BMSCs proliferation was recorded when cells were treated with 1 mM metformin. The proliferation of BMSCs in cultures exposed to 1 mM and 5 mM metformin decreased significantly after 72 hours. The significant inhibition of BMSCs proliferation treated with 10 mM metformin was observed after 48 h of culture (Figure 2(a)). The analysis of cellular activity measured by BrdU incorporation into DNA of actively proliferating cells showed a decrease of DNA synthesis after 72 h. Significant changes of BMSC proliferative activity were observed in cultures treated with 5 mM and 10 mM metformin in comparison to the control culture. BMSCs treated with 10 mM metformin showed reduced DNA synthesis from the beginning of the experiment (Figure 3(a)).

After 24 hours of propagation, no significant changes were observed in the proliferation of Balb/3T3 in control and experimental culture. However, 48-hour metformin exposure of Balb/3T3 cultures negatively influenced metabolic activity of cells. Interestingly, in Balb/3T3 culture metformin at 1 mM and 5 mM concentrations exerted comparable effects on the cell proliferation. Growth curves of these cultures had similar patterns, indicating culture restoration and increase of cell proliferative activity after 72 hours. Similarly as in BMSC cultures, the activity of cells was significantly reduced after exposure to 10 mM metformin (Figure 2(b)). BrdU incorporation assay showed that synthesis of DNA in Balb/3T3 was not altered by metformin after 24 hours of propagation. Interestingly, after 48 hours of culture, 1 mM metformin induced DNA synthesis in Balb/3T3. Cytotoxic effect of 10 mM metformin on Balb/3T3 was observed after 48 hours of culture. Reduced DNA synthesis was found in all experimental cultures after 72 hours of propagation; however, statistical significance was reached in the case of cultures treated with 5 mM and 10 mM metformin (Figure 3(b)).

### 3.3. Morphology of Cells in Cultures with Metformin

**Figure 4:**
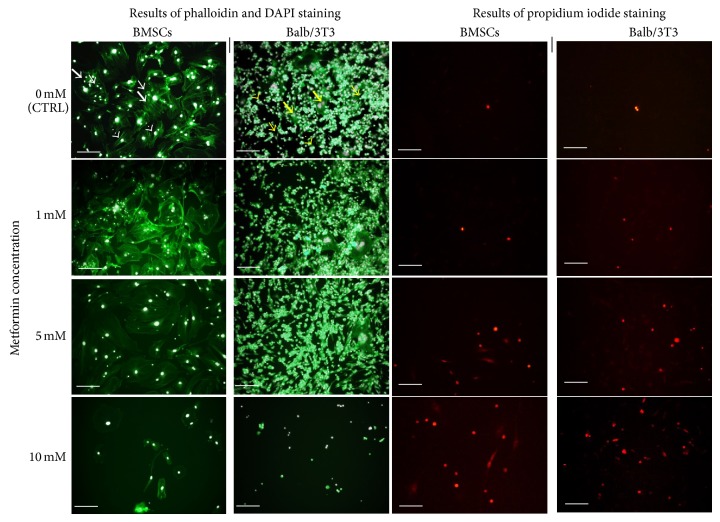
Morphology of murine BMSCs (left column) and Balb 3T3 (right column) in the control and experimental cultures. Three distinct cell types of BMSCs are indicated with white arrows: large flat cells (thick arrows), smaller fibroblast-like cells (arrows with dotted shaft), and small round cells (thin arrows). Morphotypes of Balb/3T3 cells are indicated with yellow arrows: large multinucleated cells (thick arrows), fibroblast-shaped cells (thin arrows), and small round cells (arrows with dotted shafts). Cytoskeleton was stained using atto-488 phalloidin; therefore, cellular bodies are stained in green. Nuclei stained with DAPI are visible as white dots, while dead cells visualized in the reaction with propidium iodide are stained in red. Magnification 100x, scale bar = 200 *μ*m.

Evaluation of morphological changes was performed after 48-hour propagation based on the results of cytotoxic assay (Figure 4).

**Figure 5:**
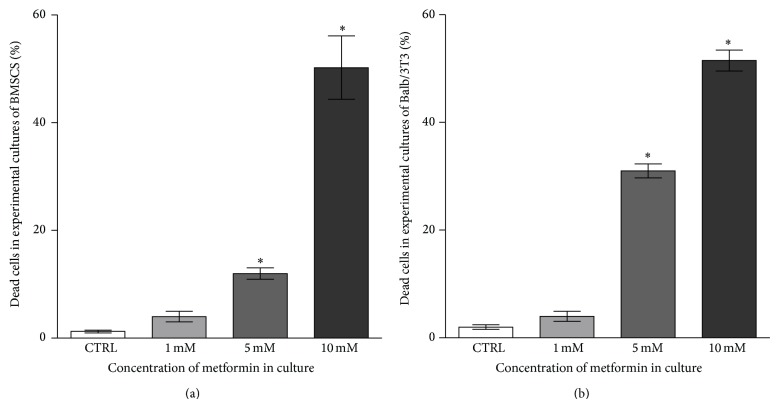
The percentage of dead cells quantified after propidium iodide staining. Calculation was performed based on the images obtained from three independent experiments. Evaluation of cell viability in cultures after 48 h of treatment with 1 mM, 5 mM, and 10 mM metformin. The number of dead cells in BMSC cultures (a) and Balb/3T3 cultures (b) increased after treatment with metformin at 5 and 10 mM concentration. Statistical analysis was performed in relation to the results obtained for control culture (no metformin). An asterisk (∗) indicates a statistically significant difference (*p* < 0.0001). Significance was determined by one-way ANOVA test.

Control culture of murine BMSCs was characterized by heterogeneous morphology. Three distinct cell types were present: population of fibroblast-like cells, with the predominance of bi- or multipolar cells, the most apparent large flat cells of irregular shape, and small cells adhering to the surface of large cells. Cytoskeleton of smaller fibroblast cells and large flat cells was well developed and nuclei were centrally localized, while the cytoskeleton of small cells was less developed, forming a thin rim around the oval nuclei. Moreover, small cells had characteristic actin projections on the edge of the cell body—lamellipodia. Small cells were more numerous in BMSC culture treated with metformin at a concentration of 1 mM, even though both fibroblast-like and large cells were still prevalent. No signs of cytoskeleton deformation or nuclei degradation were observed. Moreover, the confluence of culture treated with 1 mM metformin was similar to the control culture. The amount of dead cells visualized with propidium iodide was increasing with the metformin dose, as shown by quantitative analysis (Figure 5).

**Figure 6:**
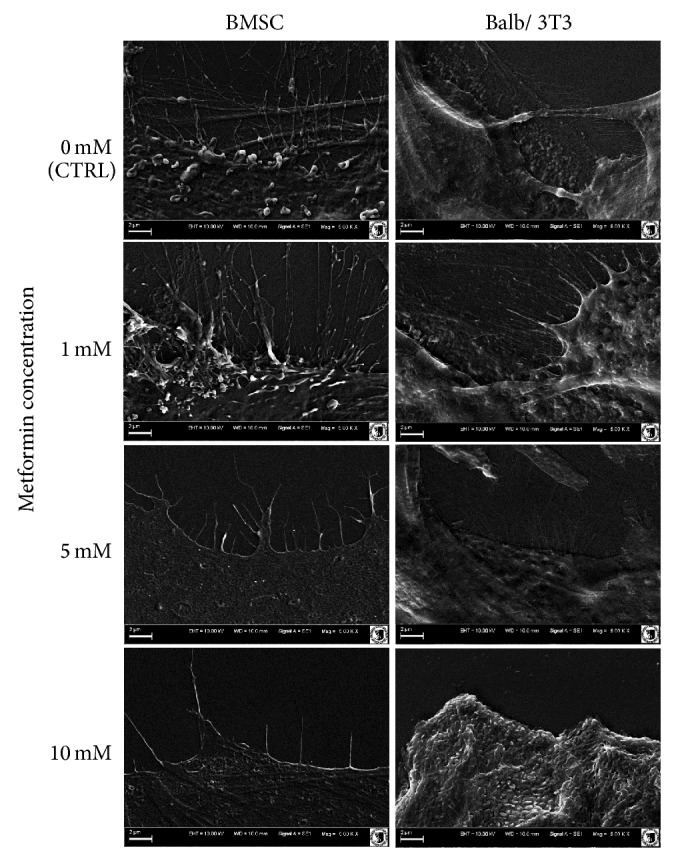
SEM analysis of cellular membrane projections. Images were captured at magnification 5000-fold, scale bar = 2 *μ*m.

Additionally, SEM analysis showed that the cells from cultures propagated in the presence of 1 mM metformin developed numerous thin cytoskeletal projections (filopodia) and secreted many microvesicles, similarly to the cells of the control culture (Figure 6). Metformin added at a concentration of 5 mM caused the enlargement of flat cells. Isolated small cells attached to the large cells were observed. In addition, cells with peripherally located nuclei were detected. The analysis of cell surface demonstrated that the projections were reduced and shortened. Significant morphological changes of BMSCs were observed when 10 mM metformin was added to the culture. The number of cells decreased, resulting in the partial loss of intracellular connections. The remaining cells in the culture with 10 mM metformin had an irregular shape and the cellular projections were underdeveloped.

Although small, oval or spindle-shaped cells were characteristic for the control culture of Balb/3T3 fibroblasts, enlarged and multinuclear cells were also present. The cytoskeleton of Balb/3T3 cells was well developed in the case of cells with a typical fibroblast morphotype or large cell body. Cytoplasmic organelles of oval-shaped cells were limited to the rim around nuclei. No signs of apoptosis were observed. The pattern of growth of 3T3/control culture was random, cells formed aggregates, next to densely and evenly arranged areas of culture. The introduction of metformin to 3T3/Balb culture at a concentration of 1 mM and 5 mM had no significant effect on the pattern of growth and morphology of cells; nevertheless, a decrease in the number of large multinuclear cells was visible. Furthermore, evaluation of cell surface showed that the cells from cultures with 1 mM and 5 mM metformin had well developed filopodia and lamellipodia. Cytotoxic effect of metformin at 10 mM concentration was apparent when cell morphology was evaluated. Cell bodies were significantly reduced and cellular debris was dominant in the image. SEM analysis showed lack of cellular projections and shrunken cell bodies.

### 3.4. Ultrastructure of Cells

**Figure 7:**
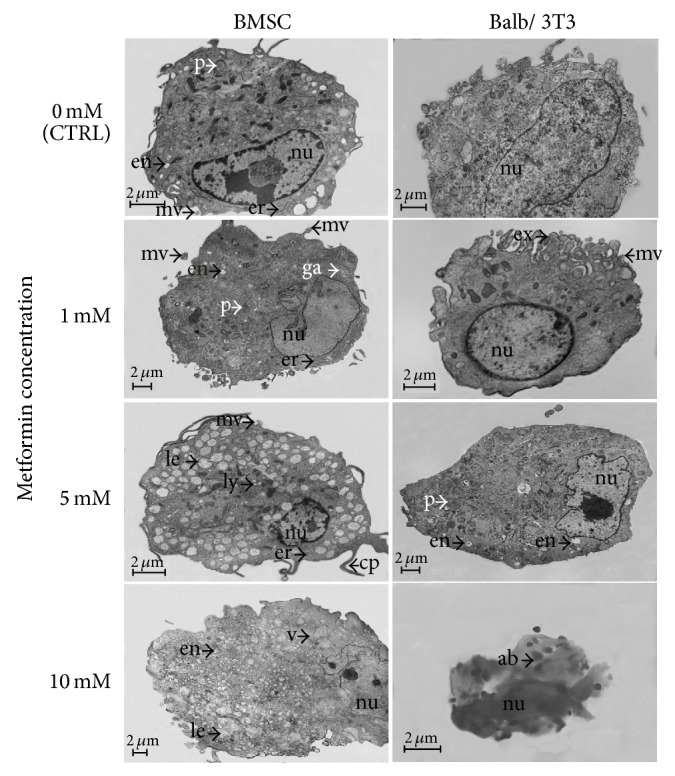
Ultrastructure of BMSCs and Balb/3T3 cells of control and experimental cultures. Scale bar = 2 *μ*m. nu: nucleus; en: endosomes; le: late endosomes; ex: exosomes; er: endoplasmic reticulum; mv: mesenchymal microvesicles; ga: Golgi apparatus; cp: cellular projections; ly: lysosomes; p: peroxisomes; v: vacuoles; ab: apoptotic bodies.

Transmission electron microscopy revealed changes in the structure and arrangement of organelles after treatment with metformin (Figure 7). In both cell types the most evident changes were related to the shape of the nucleus. The nuclei of BMSCs were malformed even when treated with metformin at the lowest concentration, while in the case of Balb/3T3, changes in the shape of nuclei were visible after treatment with 5 mM metformin.

BMSCs treated with 1 mM metformin had a better developed rough reticulum when compared to the control culture. Golgi apparatus was also more apparent. The number of endosomes and peroxisomes in BMSCs treated with 1 mM concentration was comparable to the control culture, but microvesicles were more abundant. The ultrastructural analysis of BMSCs cultured with 5 mM concentration of the drug showed an increase in the number of late endosomes and lysosomes. Long cellular projections were also characteristic of BMSCs cultured in 5 mM metformin, but microvesicles were observed sporadically. BMSCs treated with 10 mM metformin had an irregular shape, and the cytoplasm was filled with vacuoles. The initial stage of apoptotic body formation was also recorded.

A distinctive feature of Balb/3T3 cells cultured with 1 mM metformin was a ridged cellular membrane releasing microvesicles and exosomes. The addition of metformin at a concentration of 5 mM resulted in enhanced production of endosomes and peroxisomes, while the release of microvesicles was reduced. Complete damage of Balb/3T3 cells was recorded in the culture with 10 mM metformin. Microscope imaging showed only fragmented nuclei and small apoptotic bodies.

### 3.5. Analysis of Gene Expression

**Figure 8:**
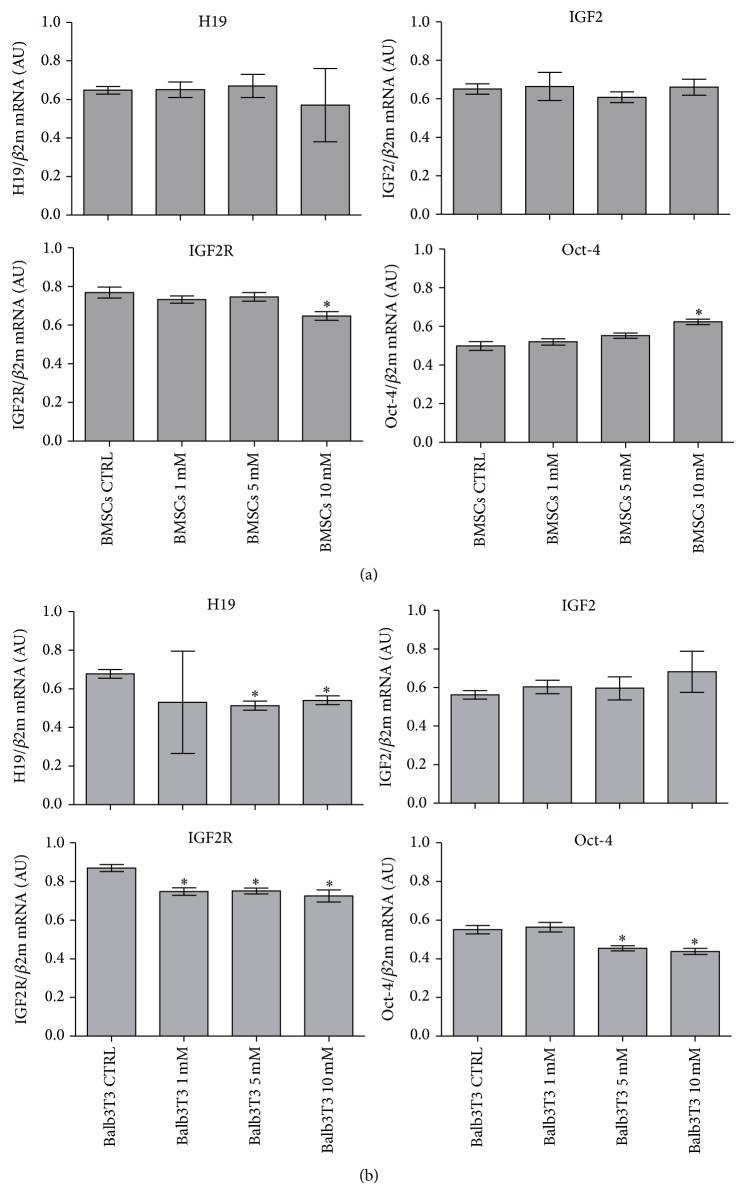
mRNA expression of H19, insulin-like growth factor 2 (IGF2), its receptor (IGF2R), and Oct-4 in BMSCs (a) and Balb/3T3 cell line (b) in control and experimental cultures. The level of expression of all genes was calculated in relation to the housekeeping gene, beta 2 microglobulin (*β*2m). Results are presented as the mean of three independent experiments ± standard deviation (±SD). Significance was determined by one-way ANOVA test (*p* < 0.05). Description of observed relationships was included in the main body of the paper.

The next stage of the study was to determine the expression of genes associated with proliferative potential of cells. Consequently, the analysis was performed on cells derived from cultures propagated for 48 hours. Quantitative analysis of transcripts revealed that the expression of H19 and IGF2 in BMSCs was not altered in the experimental cultures. The level of IGF2R transcript in BMSCs was constant in cultures with 1 mM and 5 mM metformin but decreased in the cultures with 10 mM concentration. In turn, the expression of Oct-4 gene was increased in BMSC cultures propagated with 10 mM metformin, while the transcript level in cultures with 1 mM and 5 mM metformin was not changed when compared to the control culture (Figure 8(a)).

The expression of H19 gene in Balb/3T3 cells was reduced in cultures treated with metformin; however, significant changes in transcript level, compared to the control culture, were observed only when cells were treated with 5 and 10 mM metformin. The investigated concentrations of metformin did not influence the amount of IGF2 transcript in Balb/3T3 cells; in turn, lower level of IGF2R mRNA was observed when compared to the control culture. The level of Oct-4 transcript was significantly decreased in cultures treated with 5 and 10 mM metformin (Figure 8(b)).

### 3.6. Concentration of IGF2 Protein in Culture Supernatants

**Figure 9:**
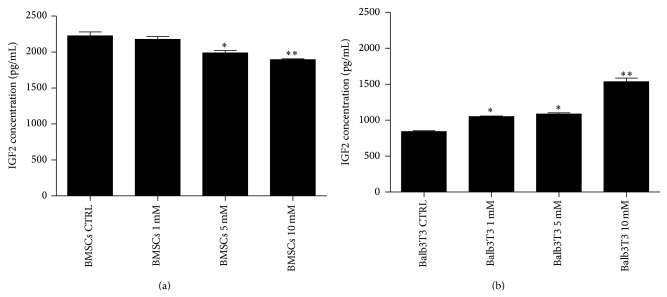
Quantitative analysis of IGF2 protein level in the supernatants after BMSC (a) and Balb/3T3 (b) cultures. Statistically significant differences were observed at *p* < 0.05 (∗) and *p* < 0.01 (∗∗).

Quantitative analysis of IGF2 concentration showed that the propagation of BMSCs with 5 mM and 10 mM metformin decreased the level of this protein. Different patterns were observed for Balb/3T3, where an increase of metformin level was positively correlated with the concentration of IGF2 protein (Figure 9).

## 4. Discussion

Currently, metformin is perceived not only as a hypoglycemic agent but also as a comprehensive medication. The administration, among many others, is recommended in medical conditions associated with metabolic disorders. However, the greatest expectations are held with the application of metformin in the treatment of cancer and activation of endogenous adult stem cells [28]. Antiaging activity of metformin combined with the potential of mesenchymal stromal stem cells would provide a perfect solution for the needs of regenerative medicine, mainly due to the fact that metformin may modulate proliferation of MSCs and promote their differentiation towards osteoblast cells. Nevertheless, a review of the literature indicates that biological effects of metformin are predominantly studied using immortalized cell lines, while studies that focus on the influence of metformin on mesenchymal multipotent stem cells are limited.

In this study, we decided to analyze the effect of metformin on the proliferative activity, morphology, and ultrastructure of two populations of cells: (i) primary cultures of bone marrow-derived multipotent mesenchymal stromal stem cells and (ii) well established fibroblast Balb/3T3 cell line. As opposed to the committed cells (here fibroblasts), mesenchymal stem cells possess the unique ability to self-renew and to differentiate into other cells of mesodermal lineages [29–32].

Antitumor effect of metformin was established for doses from 5 to 30 mM [4, 5, 7, 8, 33]. The concentrations of metformin added to cultures are well above the concentration range, in which metformin can be safely used* in vivo *[34, 35]. However, it was also proven that the concentration of metformin accumulating in the tissues might be several times higher than in blood, and thus metformin is present at a significantly higher levels in the target organs [36].

The effect of metformin on BMSCs has been investigated by Gao et al. [37] and Molinuevo et al. [38]. The study of Gao et al. [37] demonstrated that BMSCs cultured in osteogenic conditions and treated with 100 *μ*M metformin responded with an increase in proliferative activity and osteogenesis. Molinuevo et al. [38] reported that metformin induced osteogenesis of BMSCs both* in vitro* and* in vivo* and enhanced the process of bone repair in diabetic and nondiabetic rats. However, both experiments were disputed by the Jeyabalan et al. [39]. Both Gao and Molinuevo used low doses of metformin in their* in vitro* models, while we were interested in the effect of higher concentrations of metformin, that is, 1 mM, 5 mM, and 10 mM, which are the doses exerting antitumor effect. Our results demonstrated significant inhibition of BMSC proliferation rate at 10 mM concentration of metformin. The addition of 1 mM and 5 mM doses decreased proliferative activity of mouse BMSCs but only after 72 hours of propagation. Recently, Abu-Zaiton [40] showed that the proliferation of dermal fibroblasts is affected by metformin in a dose-dependent manner. Our results showed that metformin at a concentration of 1 mM and 5 mM exerted comparable effect on the proliferative activity of Balb/3T3 fibroblasts. Interestingly, fibroblast proliferative potential after application of 1 mM and 5 mM metformin was restored after 72 hours of culture. Nevertheless, the effect of all concentrations investigated significantly reduced fibroblast proliferative activity and this result is consistent with Abu-Zaiton's findings.

The influence of metformin on physiology of BMSCs and fibroblasts has been thus far predominantly studied in the context of their proliferation [38, 40]. However, these studies used lower concentration of metformin and experiments were performed independently. In our model, due to the use of two cell populations of stromal origin, but differing in the context of proliferative potential (nonimmortalized BMSCs and immortalized Balb/3T3), we could perform comparative analysis of their cytophysiological properties. Our experiment shows that proliferation activity of BMSCs and Balb/3T3 fibroblasts is significantly inhibited after exposition to 10 mM metformin. Growth of both BMSCs and Balb/3T3 was significantly influenced by metformin at 1 mM and 5 mM concentration after 72 h. The difference in proliferative potential of BMSCs and Balb/3T3 is significant, which is confirmed by the growth curves; Balb/3T3 cells attempted to restore the population despite the inhibition of metabolic activity after exposition to 1 mM and 5 mM metformin, while BMSC growth was declined.

Decrease in cell proliferation can be associated with cytotoxic effect of metformin, which has been previously observed in various cancer cell lines [4, 5, 7, 8, 33]. The results of the BrdU assay confirmed that metformin has great impact on cellular activity of both BMSCs and Balb/3T3 in a dose-dependent manner. Significant inhibition of the number of actively proliferating cells, expressed by the reduction of DNA synthesis, was noted after treatment with 5 mM and 10 mM metformin. This finding was also consistent with the results of propidium iodide staining. The percentage of dead cells increased in cultures treated with 5 and 10 mM metformin. Metformin affected physiology of the cells not only by reducing proliferating activity but also by causing morphological and ultrastructural alterations.

Undeniably, significant part of our experimental model was focused on the evaluation of morphological and ultrastructural changes of cells after metformin treatment. We argue that the analysis of cellular organization should be a key parameter when determining the effects of active agents, such as metformin, even though the evaluation of cell proliferation and gene expression becomes an important complement to such analysis. Detailed investigation of cell morphology is often neglected, due to the difficult and time-consuming techniques or is sometimes reduced to the analysis of cell shapes and growth patterns with an inverted light or fluorescence microscope, as in the papers discussed [38, 40]. To the best of our knowledge, the present study is the first report showing morphological and ultrastructural changes of BMSCs and Balb/3T3 fibroblasts under the influence of metformin.

Morphology is an important large-scale manifestation of the global organizational and physiological state of the cells [41]; therefore, we believe that the presented approach will help understand how structure and function of cells examined are interrelated. The analysis of cell morphology in the context of anticancer compounds may provide meaningful data of clinical significance, because traditional diagnosis of pathology is established on the basis of morphological findings [42].

Epifluorescence microscopy in BMSC control culture revealed the occurrence of three distinct morphotypes. Our observation corresponds with findings presented by Ren et al. [20] and Wieczorek et al. [43] who have also described three different types of BMSCs: (i) large flat cells; (ii) smaller fibroblast-like cells; and (iii) small round cells. Our results demonstrated that with increasing concentrations of metformin the number of small round cells is reduced. The study of Ren et al. [20] indicated that cultures enriched in small cells had a greater multipotent differentiation potential than the cultures with a higher ratio of large cells. Therefore, we concluded that high doses of metformin may negatively affect the “stemness” of BMSCs. Similarly, SEM analysis showed that the number of cellular projections decreased with increasing concentrations of metformin. Ultrastructural evaluation showed signs of BMSC autophagy after metformin treatment at a concentration of 5 mM, reflected in the fusion of late endosomes with lysosomes [43]. Nuclear disorganization was also characteristic of cells treated with metformin. Three types of cells could also be distinguished in the Balb/3T3 fibroblast cultures that were typical for this cell line, that is, (i) small round cells, (ii) fibroblast-shaped cells, and (iii) multinucleated large cells. Holt and Grainger [44] argued that immortalized fibroblasts may form multinucleated cells via fusion with other fibroblasts, which is typical of various pathologies such as fibrosis, cancer, aging, and foreign body response (FBR). Our results showed that the number of multinucleated giant cells is reduced in the Balb/3T3 cultures treated with metformin at a concentration of 1 mM and 5 mM. Cytotoxic effect of the highest dosage of metformin in Balb/3T3 was confirmed by means of fluorescent and scanning electron microscopy. Evaluation of cellular projections with SEM showed that the cultures treated with 1 mM and 5 mM metformin had well developed filopodia and lamellipodia. Ultrastructural analysis revealed significant alterations in cellular organization after treatment with 5 mM metformin.

Analysis of gene expression of the components of IGF2 signaling pathway showed that the expression of IGF2 gene was not affected by metformin treatment neither in BMSC nor Balb/3T3 cell line. The expression of H19 in BMSCs demonstrated that metformin did not influence the level of mRNA. What is more, the level of H19 transcript was comparable with IGF2 mRNA level, which contributes to maintaining the proliferation balance [15]. Expression of IGF2 receptor mRNA was decreased in all experimental cultures of Balb/3T3, while in BMSCs, IGF2R expression was reduced in cultures treated with 10 mM metformin. The expression of H19 gene in the case of Balb/3T3 was significantly reduced after treatment with 5 mM and 10 mM concentrations when compared to control.

Synthesis of IGF2 mRNA seems to be functioning in a constitutive manner. IGF2 is strongly involved in cell proliferation, survival, and migration [15, 45]. A decrease in the IGF2 protein contents in the supernatants collected after BMSC cultures with metformin correlated with the loss of proliferation potential of the cells. However, an increase of IGF level was positively correlated with higher doses of metformin in Balb/3T3 cell line. We believe that this phenomenon might be associated with prevention of cell death, particularly in view of a significant decrease in the expression of Oct-4 gene in Balb/3T3 cells treated with 5 and 10 mM metformin. It was shown that fibroblasts show basal expression of certain pluripotency related genes, including Oct-4, and culture conditions may affect the expression pattern of these genes [46]. Our results indicate that metformin at 5 and 10 mM concentration may decrease the transcript level of Oct-4 in Balb/3T3 cultures. In turn, the differentiation markers of BMSC linage are well established, whereas only few literature reports concern the expression of pluripotent genes including Oct-4, which is a critical transcription factor regulating self-renewal and differentiation of stem cells [20, 47]. As previously reported [48], Oct-4 expression may be induced in somatic cells in response to various stress stimuli. This thesis could be true in the case of BMSC culture, as the expression of Oct-4 was significantly enhanced after treatment with 10 mM metformin.

## 5. Conclusions

In summary, metformin introduced to BMSC and Balb/3T3 cultures at a concentration equal to 5 mM and 10 mM exerted cytotoxic effect, which was reflected in (i) a decrease of cell proliferation, (ii) increase in the incidence of cell death, and (iii) disintegration of cultures, manifested with morphological and ultrastructural changes. Undoubtedly, it is necessary to elucidate the molecular mechanisms determining the underlying effects of metformin both* in vitro* and* in vivo*; nevertheless our results demonstrate that the analysis of cell morphology and ultrastructure may provide additional information that may help to understand the complexity of metformin action. We believe that the analysis of cell morphology in the context of anticancer compounds may provide meaningful information of clinical significance.

## Figures and Tables

**Table 1:** Sequences of primers used in qPCR.

Gene	Abbreviation	Sequence 5′-3′	Loci	Amplicon length [bp]	Accession number
Beta-2 microglobulin	b2m	F: CATACGCCTGCAGAGTTAAGCA	341–362	73	NM_009735.3
		R: GATCACATGTCTCGATCCCAGTAG	413–390		

Insulin-like growth factor 2	IGF2	F: TCAGTTTGTCTGTTCGGACCG	223–243	223	NM_001122737.1
		R: TTGGAAGAACTTGCCCACG	445–427		

Insulin-like growth factor 2 receptor	IGF2R	F: GGCTGCGATCGATATGCATCT	2616–2636	106	NM_010515.2
		R: GGCCTATCTTTGCAACTCCCA	2721–2701		

H19	H19	F: AGGTGAAGCTGAAAG	2031–2045	97	NR_001592.1
		R: GCAGAGTTGGCCATGAAGATG	2127–2107		

Octamer binding transcription factor 4	Oct-4	F: TTCTGCGGAGGGATGGCATA	258–277	232	NT_039649.8
		R: GTTCTAGCTCCTTCTGCAGGG	489–469		
